# Reach and Utility of COVID-19 Information and Preventive Measures for Nomadic Populations in Massangam, West Region of Cameroon

**DOI:** 10.4269/ajtmh.21-0792

**Published:** 2022-03-02

**Authors:** Kareen Atekem, Ruth Dixon, Rogers Nditanchou, Christine Masong Makia, Marlene Ntsinda, Sapana Basnet, Elena Schmidt

**Affiliations:** ^1^ Sightsavers Research Team, Yaoundé, Cameroon;; ^2^ Sightsavers Research Team, Haywards Heath, United Kingdom;; ^3^ Catholic University of Central Africa, Institute of Policies and Social Initiatives (IPIS), Yaoundé, Cameroon

## Abstract

The separation of nomadic pastoralist settlements from settled communities is a well-known challenge to the health system. Difficulties reaching these groups contribute to inequities in their health and impact the spread or control of several diseases. COVID-19 has led to the suspension of many public health interventions in Cameroon, while preventive measures including behavior change communication have been ongoing since the onset of the pandemic. The reach and utility of these campaigns in semi-nomadic population remain unclear. This exploratory qualitative study was conducted in September to October 2020 using semi-structured interviews and focus group discussions with nomadic camp heads, and their wives to explore their interactions with communication campaigns, awareness, understanding, and acceptance of behavior change messages. The study revealed a general awareness of COVID-19 and its preventive measures and a prevailing belief that they were less at risk because their camps are isolated from the main communities, and the fact that they had never met a COVID-19 case. They perceived that the women were at lower risk because of their limited interaction outside the camps. There was a common concern regarding the transmission of COVID-19 to their cattle. Routes of communication were markets and mosques frequented by men, making access to information limited to or dependent on men. Financial constraints and lack of water were the main barriers affecting the access to and use of COVID-19 prevention measures. There is need for adaptive communication strategies especially tailored to the culture of nomadic pastoralists addressing gender dynamics of this subgroup.

## INTRODUCTION

COVID-19, a coronavirus disease caused by the novel coronavirus severe acute respiratory syndrome coronavirus 2 (SARS-COV-2) was first reported in late 2019 in Wuhan, China.^1^ The disease rapidly swept across all continents and was declared a global pandemic by the World Health Organization (WHO) in March 2020. Subsequently, many public health interventions including mass drug administration (MDA) for Neglected Tropical Diseases (NTDs) were suspended to curb the spread of the disease. Transmission of the virus was understood to be through droplets generated when an infected person coughs, sneezes, or exhales, and the WHO recommended physical distancing, wearing of a face mask, and handwashing, as key preventive measures.^2^ Within some countries, additional preventive measures such as closure of public gatherings (churches, schools, funerals, etc.), and restriction of movements both locally (lockdowns) and internationally have been implemented.

Cameroon is a country in West-Central Africa with a population approximately 25.9 million people as of 2019.^3^ The population of Cameroon is heterogenous with over 240 ethnic groups spread over the Francophone and Anglophone regions and speaking two principal languages, French and English, and over 260 local languages. The first case of COVID-19 in Cameroon was detected in early March 2020^4^ and within months, the number of cases had rapidly increased^5^; with East Region, Douala (in the Littoral Region), West Region, and Yaoundé (in the Center Region) being at highest risk for COVID-19, and prevalence greater in male sex, hypertension, and diabetes patients.^6^^,^^7^ Since the onset of the disease, the Ministry of Health (MoH) together with local authorities put forth a range of disease control measures, including a COVID-19 Surveillance and Response Strategy and subsequently a Preparedness and Response Plan^8^ for early detection of cases, contact tracing, and disease management. Emergency Operations Centers (EOC) at national and regional levels were capacitated for case detection and management. Communication messages in the form of posters, banners, radio, and other media outlets were made available to the public alongside a hotline to obtain information and report cases. Posters and banners were pasted in public places such as markets, offices, churches, and health facilities, and included information on the signs and symptoms of the disease, preventive measures, and case reporting.

In addition to multiple ethnic groups in the settled communities of Cameroon, there are a large number of nomadic populations. These are traditional groups of hunter-gatherers and pastoralists, who do not have fixed habitation and regularly move to and from the same areas.^9^ Across Africa, nomadic groups have been recognized as being hard-to-reach with health interventions because of their remoteness, mobile nature of living, and language and cultural differences with the settled communities.^10^ For example, in Northern Senegal, the utilization of malaria control interventions among nomadic pastoralists has been reported to be significantly lower than in the general population.^11^ Similarly, in Somalia, up to 90% of nomads have been reported to be out of reach of the national health services.^12^ In Cameroon, nomadic groups have been identified as a potential reservoir for ongoing transmission of onchocerciasis, a neglected tropical disease prevalent in many parts of the country.^13^ Based on the evidence from the control programs of other infectious diseases, one would expect nomadic population to be at risk of missing out on COVID-19 information and prevention measures. However, systematic data on this is currently very limited.

The study presented here was conducted in Massangam district, West Region of Cameroon, where Fulani nomads constitute close to 20% of the local population.^14^ The purpose of the study was to explore the availability of information and acceptability of behavior change communication messages to these nomadic population during the COVID-19 pandemic and lockdown.

## METHODS

### Study area.

**Figure 1.**
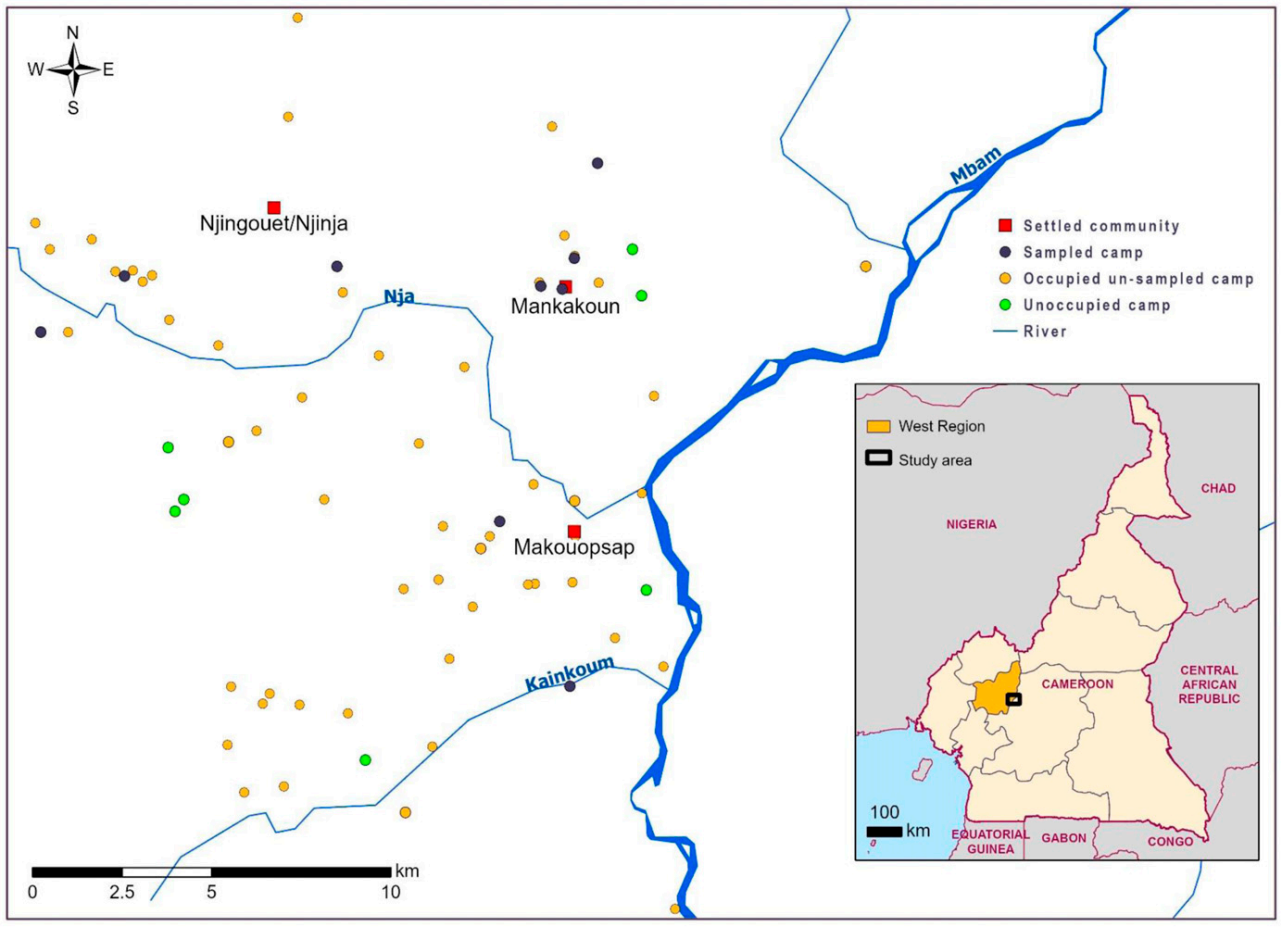
Map showing camps within the three settled communities, and camps visited under the research. This figure appears in color at www.ajtmh.org.

The study was conducted in three settled communities, Makouopsap, Makankoun, and Njinja/Njinguoet in Massangam, through which nomadic people move seasonally (Figure 1). The study was integrated within an ongoing NTD program, which provided annual MDA of ivermectin and was piloting a test and treat strategy with doxycycline within these communities. As part of the program, in September 2020, 47 encampments, where nomadic people temporarily settle were mapped in this area. The mapping showed that about 83% of the nomadic encampments were occupied by nomads at the time with the number of temporary residents per camp ranging from 2 to about 20. This population of nomads are Fulani, speaking Fulfulde dialect and known locally as “Bororo,” who migrated from other neighboring countries such as Nigeria, Chad, and Central Africa Republic into Cameroon. The majority of nomads in this area are herdsmen, who move (transhumance) with their cattle out of the main communities to riverine areas during the dry season to exploit seasonal availabilities of grazing and water. Typically, in this area, adult men move with the cattle, while the elderly, women, and children remain in the encampments. Another common pattern of movement seen in this group of people is the diurnal movement, where the male children and their fathers move with the animals early in the morning into the bushes and return to their huts at dusk. Some nomads practice additional activities such as small businesses and farming. During the mapping of the encampments, the implementing team became aware that these nomadic people had very varied access to health services available in the areas and limited information about COVID-19.

### Study design.

We conducted an exploratory qualitative study. Data were collected in September and October 2020 using semistructured interviews and focus group discussions (FGDs) with nomadic camp heads, their wives, and other male and female residents.

### Sampling.

A total of 27 people (19 men and 8 women) were selected to participate in two in-depth interviews (IDI) and two FGDs (Table 1). Participants were recruited purposefully to reflect a variety of locations and camp types.

**Table 1:** Study participants

Participants	Interviews		Sex		Total
	In-depth interviews	Group discussions	Male	Female	
Camp heads	9	1 (FGD with 10 men)	19	0	19
Camp heads’ wives	4	1 (FGD with 4 women)	0	8	8
Total	13	2	19	8	**27**

FGD = focus group discussion.

### Data collection.

Semi-structured interviews and FGDs were conducted using a topic guide and probes to facilitate the discussion. Demographic information was collected before the interview/FGDs. Since both FGDs and IDIs were held with similar profile participants, data collection only continued until reaching saturation, with no new emerging information, codes, and themes. Each interview/FGD lasted 30–45 minutes and all were audio-recorded with supplementary field notes. The interviews/FGDs were conducted in the language the participants were most comfortable (pidgin, English, French, or Fulfulde) and translated where appropriate with the help of local guides.

### Data analysis.

All audio records were transcribed verbatim and coded independently by two researchers. An inductive analysis approach guided by^15^ thematic analysis method was used to identify codes, analyze them further, and organize them into themes and subthemes. The process was iterative and comparative, comparing codes to find consistencies between the data from IDIs and FGDs. Disagreements in coding among researchers prompted reflection, and open discussions on differences in coding were held till a consensus was reached and the coding frame adapted to reflect unanimous viewpoints. This coding frame was then applied systematically to the data.

### Ethics.

This study was approved by the National Ethics Committee (*Comite National d’éthique de la Recherche pour la Santé Humaine)* in Cameroon, approval N^o^2020/01/1203/CE/CNERSH/SP. The study was explained to all participants in their preferred language. An individual written informed consent was obtained in all cases prior to participation.

## FINDINGS

### Characteristics of camps and participants.

Interviews and FGDs were conducted in nine camps. The camp populations ranged from 3 to 13 people. Smaller camps tended to include only the head of the camp, his wife, and their immediate family members (e.g., children); larger camps tended to have more extended family members, for example, siblings of the head of the camp. The majority of study participants were males (70.4%). The age ranged from 20 to 77 years with two-thirds of respondents being above the age of 30 years. All female participants were wives of the heads of the camps. The majority of camps (80%) had spring water as their source of water.

Data arising from the analysis were organized into three main themes following the objectives of the study: 1) understanding COVID-19, 2) access to information, and 3) prevention and care.

### Understanding COVID-19.

#### Disease name and causes.

Most participants said that they had heard about the new disease, which has been affecting many people. A number of people referred to the disease by the name of the virus (Corona), although quite a few struggled to call the name correctly:

*“It is colona . . . corona . . . I have forgotten how it is called. Corona bilisss. That is how I heard people calling it. Corona biliss”* (male, Camp leader, IDI)

Most participants said that they did not know what caused the disease, but many knew that it originated from China and found in many countries all over the world:

*“We live here in the bush, so we cannot know what causes people to have the disease. . . . I really do not know what causes it . . .”* (male, Camp leader, IDI)

*“I heard from the radio that it kills. . . . I hear that it is in the western world. I also know that it is present in Nigeria, America and also Douala”* (male, Camp leader, IDI)

#### Symptoms and routes of transmission.

Most participants were unaware of COVID-19 signs and symptoms, as they believed they themselves did not have it and they had not seen any cases in their camps or the wider settled communities, though many showed interest in learning more about it. Very few mentioned cough or fever as symptoms of COVID-19. A number of participants explained that the disease could be transmitted through droplets, handshakes, close proximity to an infected person, and when people move from one place to another, they can easily get the disease.

*“I heard that if someone has it, the person has fever, or can cough . . . that it is easy to transmit if an infected person doesn’t wear a mask, drops from his mouth and nose can contaminate other people.”* (male, Camp leader, IDI)

*“We can’t tell because people move a lot; and it is because people travel a lot that it can get to this place . . . the disease is usually present in places where many people move a lot. Some people can leave Yaoundé and Douala and Gabon. That’s when person to person transmission can occur easily. But here in the bush, it is difficult . . . We are isolated. It is only when some of us leave this place and go to Bafia, Yaoundé and others leave those places and come here that we can easily get contaminated.”* (male, Camp leader, IDI)

Being nomadic herdsmen, a common concern that cut across a number of the participants was potential transmission of COVID-19 to the cattle and, whether the cattle could contract the disease and become ill:

*“I just know that transmission is among humans. I cannot tell if animals can transmit the disease. Well, we are in the bush, so we cannot really tell of someone will bring the disease to the cows there or we the owners can contaminate the cows when we are sick, I do not really know.”* (male, Camp leader, IDI)

Some respondents did not believe that COVID-19 was real and even if it was, they were not worried about it. They further explained that, in their view, the disease affected only big towns and cities, and as they lived in the bush and isolated from the main communities, they were not at risk of contracting the virus.

*“The disease doesn’t exist for us here in the bush. Our only problem here is « mout-mout » (referring to the blackfly) . . . there is no corona here . . ., here it cannot enter since we are far inside the bush. I haven’t heard that there is any case of corona in any of the villages around this area. They only talk about it. But we do not have it here.”* (male, Camp leader, IDI)

Participants believed that men, who went outside the camp were more at risk of contracting the virus than women, who did not leave the camp:

*“Those of us who go out often are more likely to contact the disease. When we go out to other places and come back home, we can easily bring it here. Because we are told that if you greet someone and he has the corona virus, you will also get contaminated.”* (male, Camp leader, IDI)

Some participants expressed fear toward people who visited their camps, particularly those coming from towns:

*“I am scared of people who come to my home from towns where that disease is because you can bring it here.*” (male, Camp leader, IDI)

#### Access to information.

The main sources of COVID-19 information mentioned by study participants were markets, mosques, community gatherings (at health centers and community places), radio news and COVID-19 songs and phone calls; with some saying they first heard about the disease when they visited other towns:

*“I heard from my neighbor’s radio. He lives close by and . . . when I want to follow news, I go to his house to listen to the radio with him.”* (male, Camp leader, IDI)

*“When news about corona virus started, people were talking about it everywhere. In the mosques, in markets. Everywhere, people are talking about it.” *(male, Camp leader, IDI)

Many respondents mentioned that the information about COVID-19 was circulated through camp heads, who met to exchange information and then passed it to their wives and children:

*“Well, it is enough to meet a few camp heads and pass them the information. They will take note of it and tell the other family heads and that is how the information will go through and reach all of us.”* (male, Camp leaders, FGD)

### Disease prevention and care.

Most participants agreed that wearing face masks, handwashing, and social distancing would prevent them from catching the disease and transmitting it to others.

*“When we are going to crowded places, we cover our mouths and noses using the facemask, keep our distances with people. Even while in the mosque praying, we are far apart. When we come back home, we wash our hands with soap before touching anyone or anything in the house.” *(male, Camp leader, IDI)

Several respondents referred to the COVID-19 legislation and strict measures put in place by the government to ensure that people were following the rules:

*“A law was passed in Massangam that everyone had to have a facemask to the extent that when you did not have yours, you were caught. The gendarmes were catching people. If you didn’t wear your mask, they will catch you and put in the cell unless you paid a fine. So, everyone had to have it. So, when you leave your household and get to Massangam, you had to wear your facemask to avoid the being caught.”* (male, Camp leader IDI)

Some participants suggested that while peer pressure and behaviors of others encouraged them to adhere to the rules, others pointed out that these rather played a role in discouraging them:

*“Whenever I go to Masangam, Foumbot, people walking out of their houses, in the market and every public place wore mask. In this kind of situation, you just I have to follow their example and wear face masks every time you go out of your home.”* (male, Camp leaders, FGD)

*“I used to wear facemasks, but since no one is wearing it these days, I do not bother wearing it to go out either.” *(male, Camp leaders, FGD)

When asked about social distancing, study participants talked about being 1 m apart, 4 m apart, or just sitting far from another person:

*“When we were in the market, we cover our noses and our mouths. And when we are among people, we give a distance of 1 meter with one another also, when discussing with people . . .” *(male, Camp leader IDI)

Respondents also knew about the importance of self-isolation, when experiencing symptoms of the disease:

*“If someone has it, they will keep him away from other people. They said in the radio that sick people should be isolated from others. When you get it, they will lock you at your place, you stay at home to avoid spreading it to other people.” *(male, Camp leader, IDI)

Some people were aware of vaccination as a way to prevent COVID-19; others thought there are medicines, which could be given to prevent the spread, and many referred to God and praying as a way to help protect their families and communities:

*“It is only through using drugs; they can be given drugs, so they do not get sick . . . And if it comes, it will be good for people to get vaccinated.” *(male, Camp leader IDI)

*“I am asking God to help us here not to get the disease.” *(male, Camp leader FGD)

When asked about challenges in following COVID-19 preventive measures, study participants mentioned two main barriers. First, their ability to pay for face masks, soap, or hand sanitizers, although some participants said that they were given face masks for free when they visited the hospital:

*“The first one [facemask] is quite expensive, I bought it for 500frs [approximately 1US$].” *(male, Camp leaders, IDI)

Another important barrier that was raised was the lack of water. Most participants said that their main source of water, which they use for drinking, laundry, and other domestic needs, was either spring or stagnant water, and they had to cover long distances to fetch it. At times, because of seasonal variations, these streams and dug-up wells dry up, which severely affect the availability of water:

*“During the dry season, water disappears but it remains during the rainy season. We bath, cook, wash hands, drink it, wash our clothes with the water. We do everything with it. Water is always there during the rainy season but in the dry season, it dries up.” *(male, Camp leader, IDI)

*“I dug a place near the marshy area further in the bushes. We carry water when we want to bath and wash our hands too. During the dry season, the water disappears but it remains during the rainy season.” *(male, Camp leader, IDI)

## DISCUSSION

This is one of the first studies that explored the experiences of a nomadic population and their access to information and preventive measures during the COVID-19 pandemic. Findings contribute to the existing literature gaps on the opportunities and challenges of targeting hard-to-reach populations with health interventions, information, and services.^11^^,^^12^^,^^16^ Available evidence shows that these communities experience a range of geographical, social, cultural, and linguistic barriers in accessing essential services. It is critical that these barriers and their drivers are well understood and addressed on the pathways to achieving ambitious global targets of Universal Health Coverage and Leave No One Behind agenda.^17^

Our findings show that the nomadic population under study had a good understanding of COVID-19 control measures, which reflects the efforts of the government behavior change campaigns, including those that focused specifically on the nomads. Study participants had information about the routes of transmission and how to control the spread of the disease but were less aware of the virus and its origin, which probably reflects the novelty of the virus in Africa compared with other more familiar infectious diseases, such as onchocerciasis.^18^ The reliance of women (who do not often leave the encampments) on men for information creates a gap of knowledge among this group of people where some of the camp heads forget to pass on the information to their wives, and where women having access to protective measures is subject to the men’s judgment and agreement.

The knowledge of COVID-19 symptoms was limited and particularly there was a lack of awareness that the disease could be asymptomatic.^19^^–^^21^ Fear of COVID-19 was not generally present in the camps with only a few people talking about it and associating it primarily with visitors from towns. Rather, many people in these encampments were more afraid of being fined or arrested for not following the prevention measures than of COVID-19 itself. Furthermore, a greater fear was expressed toward their cattle contracting and dying of the disease. This is explainable as cattle rearing is their livelihood and source of income. Contrary to Arora et al.,^22^ most coronaphobia is associated to the unending uncertainties of the virus, unforeseen reality, the need of acquiring new practices and avoidance behavior, loss of faith in health infrastructure, contraction of COVID-19 by head of states, cautionary statements from international bodies, and infodemia.

Low perceptions of the risk of the disease were found to be related to social arrangements and nomadic lifestyles. For example, women were thought to be less at risk of contracting the virus compared with men, as they never left the camps. The risk was also thought to be lower as their camps were far away from other communities and particularly urban areas. Our findings suggest that people are less aware of the diseases and their risks if they are not exposed to disease cases in their immediate environment, and if community members are aware, they are more likely to take actions to prevent the disease reducing their susceptibility to infection.^23^

The study contributes to a body of evidence, which suggests that information and communication strategies targeting traditional and indigenous population groups should be tailored to their specific culture and living arrangements,^24^ such as information on human-to-cattle transmission; messaging content and style incorporating the idea of information being taken back to women; and the idea of asymptomatic infection awareness and prevention measures not just being for people who go into town but also for those in the encampments who have not gone to town. Our findings suggest that although modern communication technologies, such as radios and mobile phones are being used by some of the nomads, many continue to rely on more traditional communication approaches, such as community meetings, social gatherings, markets, and mosques. This is particularly important to take into account in communicating messages during COVID-19 lockdowns, which rely heavily on limiting social interactions.

The results from this study also show that the challenges affecting the access of nomadic communities to prevention and care for many diseases^10^^,^^13^^,^^25^ are also true for COVID-19, with remote location, financial constraints, and poor access to water and sanitation being predominant. It is thus critical that future COVID-19 prevention measures account for these challenges and make specific provisions to address them.

## LIMITATIONS

This is a small-scale study with a purposefully selected sample of camps and participants. The majority of our respondents were men, heads of the camps, and younger people, who tend to go out more frequently and be more exposed to information about COVID-19. Our sample did not include too many older people and particularly older women and we cannot say whether their knowledge of COVID-19 was as good as the knowledge of men heading the camps. It is also important to note that we conducted our study soon after a large government information campaign, which provided information on handwashing and hand sanitizers. Therefore, it is likely that the responses of our participants were influenced by the information received during the campaign.

## CONCLUSION AND RECOMMENDATIONS

The nomadic population in this study area were aware of COVID-19 disease, and its preventive measures, although information on disease symptoms was limited. Information was mostly available to this group of people through their local channels such as marketplaces and gatherings, and men turn to be more informed than their women as they leave the camps to these places. Challenges such as finance and inaccessibility to constant water supplies hinder the effective practices of COVID-19 measures in this group.

Key recommendations to better reach this group of people include the following:

Firstly, identifying the main routes of communication and following their laid down cultural values of going through camp heads. Secondly, with the existence of patriarchal control of information, we recommend identifying ways to reach nomadic women for more gender balance access such as inclusion of female community drug distribution (CDDs), target women association, and messages need to be tailored to explain what the risks are.

Furthermore, as cattle rearing being the main activity of the nomads and following the huge concerns raised by the nomadic population about transmission of the disease to their cattle, policy response measures should also seek to address any cross-contamination between humans and animals, and messages addressing this concern.

To address the issues of water supply and financial constraints in acquiring face masks, we recommend that provision of water and face mask to this group should be prioritized especially as many countries are at the point of the second COVID-19 wave, including Cameroon. Where possible hand sanitizers should be provided.
